# Conceptual quantitative systems pharmacology framework for supporting clinical trial design in organ-specific autoimmune and rare diseases

**DOI:** 10.3389/fimmu.2025.1714438

**Published:** 2025-12-12

**Authors:** Zhengwu Sun, Yalin Xi, Xiaoyan Lan

**Affiliations:** 1 Department of Clinical Pharmacology, Dalian Municipal Central Hospital, Central Hospital of Dalian University of Technology, Dalian, China; 2 Department of Neurology, Dalian Municipal Central Hospital, Central Hospital of Dalian University of Technology, Dalian, China

**Keywords:** quantitative systems pharmacology (QSP), clinical trial simulation (CTS), organ-specific autoimmune diseases, rare diseases, mechanistic modeling

## Abstract

Organ-specific autoimmune and rare inflammatory diseases present significant challenges for clinical trial design due to profound patient heterogeneity, small population sizes, and complex tissue-specific pathophysiology. To address these hurdles, this study proposes a conceptual Quantitative Systems Pharmacology (QSP) framework tailored to support end-to-end clinical trial design in this high-need area. The framework integrates multi-source data, including preclinical, omics, and real-world evidence, via Bayesian methods to inform prior distributions for model parameters. A core multiscale mechanistic model links intracellular signaling, cellular dynamics, and tissue-level pathology to simulate disease progression and drug effects. Virtual patient populations are generated by sampling from Bayesian posteriors, capturing real-world biological heterogeneity. These cohorts then undergo in silico clinical trial simulations to evaluate and optimize key design elements, such as dosing regimens, endpoint selection, patient stratification, and adaptive strategies, prior to real-world implementation. By providing a structured, disease-agnostic workflow, the framework enables rational decision-making for dose optimization, biomarker identification, and patient enrichment. It addresses critical bottlenecks in drug development for these complex diseases, offering a powerful tool to de-risk trials and improve the efficiency and success rate of clinical development programs.

## 1 Introduction

Organ-specific autoimmune and rare inflammatory diseases are characterized by immune system dysregulation that targets specific tissues or organs, leading to chronic inflammation, tissue damage, and functional decline (1). This spectrum encompasses classic autoimmune diseases, such as autoimmune hepatitis and primary biliary cholangitis, which are primarily mediated by adaptive immune responses, as well as autoinflammatory diseases, including certain monogenic periodic fever syndromes, which are driven by dysregulation of the innate immune system (2). The latter often represent some of the rarest conditions, where extreme patient scarcity renders conventional trial design virtually impossible (3, 4). The high failure rates in clinical trials for these diseases can be attributed to several fundamental challenges. These include profound patient heterogeneity, tissue-specific pathology, and small population sizes, which collectively complicate therapeutic development (5, 6). In practice, these challenges translate into critical obstacles such as reduced statistical power due to small, heterogeneous cohorts, the use of variable or subjective clinical endpoints, and the masking of drug efficacy by pathophysiological heterogeneity (7–9).

Quantitative Systems Pharmacology (QSP) offers an integrative modeling framework that combines systems biology with pharmacological modeling to simulate drug-disease interactions in a mechanistic, data-informed manner (10, 11). The application of QSP is particularly suited to inflammatory diseases given their underlying pathophysiology, which is driven by dysregulated biological networks operating across multiple scales. These range from intracellular signaling pathways, such as JAK-STAT and NF-κB cascades, and cytokine-mediated intercellular communication, to tissue-level injury and systemic clinical manifestations (12). Traditional reductionist approaches often fail to capture the emergent behaviors and feedback loops inherent in such networks. In contrast, QSP is designed to integrate these disparate scales within a unified computational framework, enabling the simulation of how intervention at a specific molecular target, for instance with a biologic therapeutic, propagates across the system to ultimately modulate clinical outcomes (13). Such a mechanistic, systems-level perspective is essential for de-risking drug development in highly complex disease settings (14).

This capability is reflected in the progressive operationalization of QSP within immunology and rare diseases to address translational research questions (10, 15). However, its development and application in the specific context of organ-specific autoimmune and rare diseases encounter distinct hurdles. These include extreme data sparsity, intricate tissue-level pathophysiology, and a lack of standardized modeling approaches specifically adapted to these conditions. To address these identified gaps, this study proposes a conceptual QSP framework specifically designed to navigate these challenges and to provide a structured, end-to-end workflow for supporting clinical trial design in this high-need area.

## 2 Proposed conceptual QSP framework

### 2.1 Overall framework workflow

**Figure 1:**
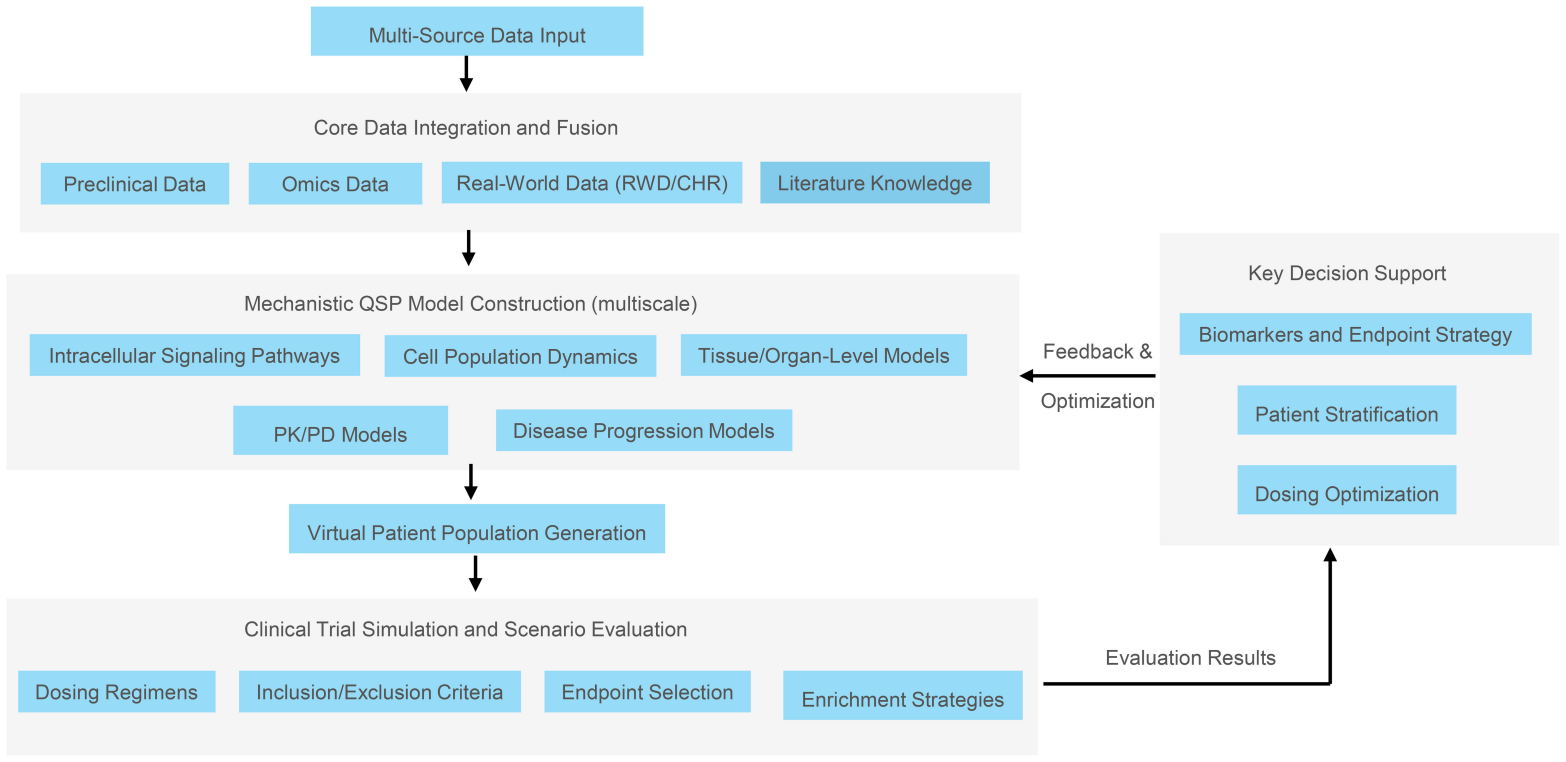
Overall QSP workflow for optimizing trial design in organ−specific autoimmune and rare diseases. The process iterates through: (1) multi-source data integration (preclinical, omics, RWD, literature); (2) multiscale modeling (signaling to cells to tissue to disease progression and PK/PD); (3) virtual patient generation via Bayesian sampling of key parameters to reflect heterogeneity; (4) in silico clinical trial simulation comparing dosing, endpoints, inclusion/exclusion, enrichment, and adaptive strategies; (5) feedback to refine assumptions and designs.

This framework provides an end-to-end in silico workflow to support decision-making from preclinical to clinical stages. Its core is an iterative, cyclic process (as depicted in **Figure 1**), beginning with the integration of multi-source data, progressing through multiscale mechanistic modeling to generate virtual patient populations, and culminating in clinical trial simulations to optimize trial design. The simulation results can be fed back to refine the models and hypotheses.

### 2.2 Core data integration

To address the core challenge of data sparsity in rare diseases, the framework employs a multi-source data fusion strategy. Data sources include preclinical data (*in vitro*, animal models), omics data (transcriptomics, proteomics), real-world data such as electronic health records and patient registries, and literature-based knowledge. The integration methodology utilizes advanced statistical techniques, such as Bayesian methods, to translate the aforementioned data into informative prior parameters for the models. This strategy is particularly critical for rare diseases, as it combats extreme data scarcity by transforming fragmented sources into a cohesive knowledge base for model construction, directly addressing the challenge of building credible models without large, homogeneous datasets.

### 2.3 Multiscale mathematical modeling

The core of this framework is a mechanism-based mathematical model designed to link systemic immune dysregulation with tissue-specific pathology, integrating multiple biological scales—including intracellular signaling pathways (e.g., IL-6, TGF-β), cellular population dynamics (T cells, B cells, fibroblasts), and tissue/organ-level pathological processes (fibrosis, functional impairment). This multiscale integration directly captures organ-specific disease manifestations.

From a technical perspective, the modeling approach typically employs systems of differential equations or agent-based simulations to characterize the dynamic interactions between immune system components and target tissues. These models are calibrated through the integration of published data, experimental results, and structured expert knowledge. Representative applications of QSP modeling components and associated candidate biomarkers across a range of organ-specific autoimmune and rare diseases are summarized in **Table 1**.

**Table 1:** Scope of organ-specific autoimmune and rare diseases and relevant QSP modeling components.

Disease/condition (organ-specific)	QSP modeling components	Candidate biomarkers/endpoints	Ref(s.)
Liver: Autoimmune hepatitis (AIH), Primary biliary cholangitis (PBC)	Immune–liver interaction modules; cytokine dynamics; drug PK/PD	ALT, AST, GGTP (liver injury markers)	(28, 58, 59)
Kidney: Lupus nephritis (LN, within SLE)	Autoantibody and complement pathways; renal involvement modules	Anti-dsDNA, C3, C4; proteinuria, eGFR	(19, 60)
Skin & joints: Psoriasis/Psoriatic arthritis (PsA)	IL-17/IL-23 pathway; cytokine–cell interactions; therapeutic PK/PD	PASI, joint counts, IL-17/IL-23 levels	(18, 61)
Gut: Crohn’s disease (CD)	Interleukin network dynamics; QSP model of rhuIL-10; simulation of therapeutic strategies	IL-10, IL-6, IL-1β; clinical indices	(31, 62)

### 2.4 Virtual patient population generation

Patient heterogeneity is reflected by sampling key parameters (e.g., cytokine baselines, immune cell activity, genetic background) from posterior distributions learned via Bayesian inference. Formally, this process can be expressed as:


p(θ|D)∝p(D|θ)p(θ)

where p(θ) is the prior distribution of model parameters, p(D|θ) the likelihood of observed data given parameters, and p(θ|D) the posterior distribution. Virtual patient i is then sampled as:


$$\theta_{i}\sim p(\theta |D),i=1,2,\ldots ,N$$

Here, each θi represents a distinct set of biological parameters for an individual virtual patient.

This approach is essential for addressing two key challenges in clinical development: first, profound immune heterogeneity, as it ensures that the virtual cohort encompasses the diverse biological states observed in real-world populations; and second, the scarcity of longitudinal biomarkers, by enabling the simulation of candidate biomarker dynamics prior to costly clinical validation.

By sampling parameters from biologically plausible distributions, the virtual cohort captures heterogeneity at its mechanistic source, the level of individual pathophysiology, reflected in variations such as signaling network activity or immune cell recruitment dynamics. This offers a more fundamental representation of diversity compared to that captured solely through broad clinical phenotypes. As a result, the framework supports the in silico simulation of clinical trials that incorporate a broader spectrum of biological profiles, including patient subpopulations often excluded by conventional enrolment criteria. By evaluating trial designs in these more representative virtual cohorts, the framework serves as a critical tool for assessing and improving the generalizability of real-world trial outcomes before patient recruitment begins (16, 17).

### 2.5 Clinical trial simulation and scenario evaluation

This is the application output of the framework, used to directly optimize and evaluate clinical trial protocols. The process involves subjecting the generated virtual patient population to different “virtual clinical trial” designs. Evaluation content includes comparing the probability of success, statistical power, and required sample size across different trial protocols such as dosing regimens, dosing frequency, inclusion/exclusion criteria, composite endpoints, and enrichment strategies. The value of this approach lies in allowing the exploration of adaptive designs (e.g., Bayesian dynamic randomization), optimization of dose escalation schemes, and evaluation of the relationship between surrogate endpoints (e.g., biomarkers) and long-term clinical efficacy without exposing real patients to risk. Thereby, it provides ethical and efficient guidance for trial design in diseases like Crohn’s disease and autoimmune hepatitis.

Within this framework, the treatment effect size is estimated by quantifying differences in clinical outcomes, such as changes in disease activity scores or responder proportions, between the virtual intervention and control arms. This simulated effect size, together with its variability across the virtual cohort, is subsequently utilized to compute statistical power and predict the probability of trial success for a specified design.

When evaluating the comparability of this approach to actual clinical trials, it is important to acknowledge both the purpose and inherent limitations of simulation-based methods. A well-calibrated QSP framework is generally not intended to precisely predict the absolute effect size observed in a future clinical trial, as real-world outcomes may be influenced by unmodeled biological variability, environmental factors, or differences in the enrolled patient population. Rather, the principal strength and comparative utility of the framework reside in its ability to robustly evaluate relative effect sizes and qualitatively rank different trial scenarios. By establishing a controlled in silico environment in which extraneous variables remain constant, the framework enables reliable comparison of strategic alternatives. For instance, determining whether an enriched population strategy produces a greater effect size than a broad recruitment approach, or whether Dose B demonstrates superior efficacy to Dose A. This capacity to systematically prioritize the most promising trial designs prior to substantial resource investment represents the fundamental value of this methodology.

### 2.6 Addressing key challenges in organ-specific autoimmunity

While grounded in established QSP principles, the present framework is specifically designed to address the distinctive challenges inherent in drug development for organ-specific autoimmune and rare diseases. It offers targeted strategies to overcome three major bottlenecks in this domain.

To address the challenge of immune heterogeneity and small patient populations, the framework employs Bayesian virtual patient generation to create in silico cohorts that mirror real-world biological diversity. This capability enables the statistical evaluation of trial designs across a large simulated population, facilitating the identification of effective patient enrichment strategies and the quantification of statistical power for adaptive designs that are critical for success in small, heterogeneous cohorts.

A key feature that distinguishes this framework from models of systemic autoimmunity is its explicit incorporation of tissue-restricted pathology within the multiscale model. The model integrates mathematical representations of key tissue-level processes, such as the activation of tissue-resident fibroblasts, collagen deposition, and parenchymal cell death. This ensures that therapeutic effects are assessed not only against systemic immune biomarkers but also against the ultimate functional and structural outcomes in the target organ, such as liver fibrosis in primary biliary cholangitis or skin plaque formation in psoriasis.

To combat the scarcity of longitudinal biomarkers, the framework serves as a rational platform for in silico biomarker discovery and evaluation. By simulating the full pathophysiological cascade, the model generates dynamic trajectories of numerous mechanistic variables, including local cytokine concentrations within virtual tissue compartments and levels of immune cell infiltration. These simulated profiles can be cross-referenced with sparse clinical data to identify the most promising candidate biomarkers for subsequent prospective validation, thereby de-risking and strategically guiding biomarker development for clinical trials.

## 3 Application of the framework to key drug development decisions

### 3.1 Dose optimization and selection

Dose optimization is particularly challenging in autoimmune and rare diseases due to narrow therapeutic windows and non-linear immune responses. The framework application involves QSP models that link drug PK to dynamic target engagement, downstream signaling pathways, and clinical biomarker changes. By incorporating physiological and pathological states, these models can simulate dose-response relationships under a range of conditions. A case example includes moderate-to-severe psoriasis, where a QSP model of certolizumab pegol integrated physiologically-based pharmacokinetics (PBPK) and systems biology approaches within a virtual population to compare 200 mg and 400 mg dosing schemes. This helped reproduce clinical efficacy patterns and revealed response variability across patient subpopulations, supporting dose optimization strategies (18). In the study of systemic lupus erythematosus (SLE), a QSP model linking anifrolumab’s pharmacokinetics to SLE’s type I interferon pathway was developed, using data from the TULIP-1/2 trials. With the 21-IFNGS as a covariate, the model showed anifrolumab 300 mg achieved rapid, sustained 21-IFNGS neutralization, while 150 mg had weaker, delayed effects. Simulations confirmed 300 mg was associated with clinical efficacy, and 150 mg sufficed for patients with reduced infection risk (19). In rheumatoid arthritis (RA), tofacitinib’s (oral JAK inhibitor) dose optimization relied on preclinical-clinical PK/PD alignment. Preclinically, mouse CIA models showed efficacy depended on JAK1 heterodimer inhibition (not JAK2 homodimers) and total daily exposure (Cave50–100 nM), with no need for continuous inhibition. Clinically, pooled Phase 2 RA data (1–15 mg BID) confirmed ED50 3.5 mg BID and Cave50–40 nM. Cave (not Cmax/Cmin) drove efficacy, with preclinical-clinical Cave50 differing by 2-fold, guiding clinical dosing (20). An overview of how QSP contributes to optimizing dose selection, sample size, endpoints, stratification, and adaptive design is provided in **Table 2**.

**Table 2:** Clinical trial design elements and how QSP contributes to optimization.

Trial design element	Traditional challenge	QSP contribution	Illustrative example
Dose selection	Narrow therapeutic windows, nonlinear immune response	Simulate PK/PD and immune pathway engagement to identify optimal dose ranges	QSP model of certolizumab pegol in psoriasis patients, used to optimize dosing and predict efficacy differences (18)
Sample size and power	Small, heterogeneous patient populations reduce statistical power	Virtual patient cohorts to evaluate required sample sizes under different assumptions	QSP model in Crohn’s disease, simulating cytokine dynamics to test different therapeutic strategies and guide sample size design (62)
Endpoint definition	Subjective, invasive, or slow-changing clinical outcomes	Identify surrogate biomarkers mechanistically linked to efficacy	Anifrolumab study, combining PBPK modeling and transcriptomics to predict tissue drug concentration and receptor occupancy, proposed as surrogate efficacy endpoints (21)
Patient stratification	Response heterogeneity leads to trial failure	Generate subpopulations based on baseline cytokine/immune profiles	Anti-IL-6 PBPK/QSP model, showing variability of CYP enzyme activity under different IL-6 levels, suggesting stratification can improve efficacy prediction (24)
Adaptive design strategies	Logistical and regulatory uncertainty	Test adaptive randomization, enrichment schemes in silico before implementation	Budesonide study, combining *in vitro* dissolution with PBPK modeling to simulate PK performance under different conditions, reducing uncertainty in adaptive trial design (63)

### 3.2 Biomarker and endpoint strategy

Defining endpoints in trials for these diseases is often difficult due to subjective assessment, long progression timelines, or invasive measurement requirements. The framework application offers a rational approach to identify biomarkers that are mechanistically linked to therapeutic action. Model outputs can be cross-referenced with omics and clinical data to nominate candidate surrogate endpoints. A case example includes systemic lupus erythematosus, where a PBPK model of anifrolumab integrated with transcriptomics-based IFNAR1 expression predicted receptor occupancy across tissues. This approach identified IFNAR1 occupancy as a mechanistically linked biomarker, supporting endpoint definition and informing Phase III trial design (21). Furthermore, the framework also clarifies clinically prevalent non-universally pathogenic biomarkers. For instance, in primary Sjögren’s syndrome (pSS), antiphospholipid antibodies (aPL) are present in 34% of patients but correlate with hypergammaglobulinemia (not thrombotic events). By integrating aPL-related signaling and immune infiltration data, the framework distinguishes mere serological findings from true pathological drivers (22). This multi-modal approach further aids RA endpoint optimization. A study using the framework integrated transcriptomics and machine learning (WGCNA, LASSO, Random Forest) to validate a 4-gene metabolic signature (AKR1C3, MCEE, POLE4, PFKM). Linked to RA’s core metabolism and immune infiltration, this signature serves as a potential surrogate endpoint, resolving the limitations of traditional assessments (23).

### 3.3 Patient stratification and enrichment

Heterogeneity in patient response is a major cause of trial failure in immune-mediated diseases. The framework application enables QSP models to simulate diverse patient subpopulations by varying baseline parameters such as cytokine levels, immune cell activity, and gene expression profiles. Coupled with machine learning, these models help identify predictive biomarkers and optimize stratification strategies. A case example includes immune-mediated inflammatory diseases, where a PBPK model incorporating literature-derived IL-6 levels constructed virtual patient populations to assess therapeutic protein drug interactions. The model demonstrated that heterogeneity in baseline cytokine levels leads to substantial variability in drug exposure, highlighting the importance of patient stratification (24). In RA, the MAPEL algorithm used virtual populations calibrated to rituximab trial data to identify a pre-treatment type I interferon signature as a key differentiator of response. The model suggested that IFNβ’s anti-inflammatory effects may counteract rituximab’s efficacy, directly leading to a predictive biomarker panel for patient stratification (25). For highly heterogeneous diseases like SLE with sparse data, Boolean network models offer a less data-intensive approach. By simulating varied patient pathways, they can identify drug targets and, crucially, stratify responders from non-responders, forming a foundational step for rational enrichment strategies (26).

## 4 Discussion

### 4.1 Summary of findings and conceptual advancements

Positioned within the evolving landscape of QSP, this study builds upon established methodologies, while addressing a critical gap in the field. Existing QSP models have demonstrated considerable value in mechanistic exploration, PK/PD prediction, and dose optimization (27), yet a generalized, systematic framework for end-to-end clinical trial design in organ-specific autoimmune and rare diseases has remained lacking (28). The framework proposed herein fills this void by offering a disease-agnostic structure that integrates multi-source data, virtual cohort generation, and in silico trial simulation within a cohesive workflow. Its novelty lies not in introducing the first QSP model, but in providing a standardized conceptual blueprint to improve the efficiency, reproducibility, and adoption of QSP in this particularly challenging domain.

By synthesizing multi-scale data spanning intracellular signaling, cellular dynamics, tissue-level pathology, and clinical outcomes, the framework enables the generation of biologically realistic virtual patient populations that reflect real-world heterogeneity (16, 29). This capability supports the *a priori* simulation and rigorous evaluation of diverse clinical trial scenarios, directly addressing the central challenges outlined in the introduction. As a result, the framework provides a rational strategy to optimize dosing regimens, identify predictive biomarkers for patient enrichment, and design more efficient trials (30), thereby de-risking drug development in these high-need therapeutic areas.

### 4.2 Generalizability, broader implications, and clinical translation

The proposed framework demonstrates strong generalizability, underpinning its potential for broad clinical translation across organ-specific autoimmune and rare autoinflammatory diseases. Its core computational structure, including integrating multi-source data, multiscale modeling, virtual population generation, and trial simulation, is intentionally agnostic to specific pathophysiology. This design allows the framework to be adapted to different diseases through the incorporation of relevant mechanistic knowledge and patient-level data, providing a reusable template for studying conditions with shared immunological features but distinct tissue manifestations.

The generalizability of the framework operates on two complementary levels. Across diseases within the same domain, reusable workflow components, such as models of T cell activation or cytokine signaling networks including IL-6 and IL-23, enable knowledge transfer between conditions (31). For instance, mechanistic principles established for modeling skin fibrosis in systemic sclerosis can be adapted to simulate lung fibrosis in idiopathic pulmonary fibrosis by adjusting tissue-specific parameters and damage readouts (32). Beyond disease-specific applications, the same calibrated framework supports a range of critical development decisions, including optimization of combination therapies, development of biomarker-informed companion diagnostics, and life-cycle management strategies such as extrapolation to new patient populations (33). In this context, the framework serves as a “development compass” for therapeutic innovation in rare and complex immune diseases. While it does not replace clinical trials, it systematically de-risks the development process by providing mechanistically grounded, quantitative priors for key design elements, ultimately increasing the probability of success and improving the efficiency of resource allocation throughout the drug development lifecycle (34).

### 4.3 Critical appraisal of challenges and translational barriers

While QSP holds considerable promise, a balanced evaluation must also consider its current limitations and the barriers hindering its broad adoption in drug development. These challenges span methodological, practical, and translational dimensions. Methodologically, QSP faces inherent tensions between biological fidelity and practical applicability. Highly detailed “bottom-up” models, though mechanistically insightful, are frequently constrained by parameter uncertainty due to sparse data, resulting in wide prediction intervals and compromised reliability (35, 36). In contrast, more empirical “top-down” models may improve short-term predictability at the expense of mechanistic transparency and generalizability. These issues are exacerbated by a reproducibility crisis, as models are often published without adequate documentation, code, or accessible data, impeding independent validation and reuse (37). Moreover, the scarcity of high-quality longitudinal data in rare diseases remains a fundamental limitation, often restricting model calibration and evaluation to retrospective, heterogeneous datasets (38).

From a practical standpoint, translating QSP into actionable decision-support tools remains challenging. A notable barrier is the communication gap between modelers and key stakeholders such as clinicians and regulators, who may perceive complex models as opaque “black boxes,” undermining trust and adoption (39). Effectively conveying model assumptions, uncertainties, and contextual relevance is thus as vital as the technical modeling process. Additionally, the substantial resources required for QSP development often conflict with the accelerated timelines and limited budgets typical of drug programs, raising valid concerns regarding return on investment, particularly in early-stage research involving novel targets (40).

While successes in dose optimization and biomarker identification are documented, other cases, such as certain models in complex diseases like Crohn’s disease, have yielded deep mechanistic understanding yet struggled to accurately predict clinical outcomes for novel therapeutic mechanisms (41). Such limitations often arise from oversimplified representations of disease heterogeneity or incomplete pathophysiological knowledge, underscoring that model predictive performance is intrinsically tied to the quality and completeness of the underlying biological data (42).

Although the proposed framework is designed for adaptability, its application to new disease contexts remains non-trivial and resource-intensive. Each adaptation demands substantial investment in disease-specific data acquisition, model recalibration and validation, and often the development of new mechanistic modules to capture unique pathological features (43). Therefore, while generalizability represents a key strength, its realization depends on focused and substantial effort, suggesting that QSP should be strategically deployed in high-value, high-uncertainty development scenarios rather than indiscriminately applied.

### 4.4 Future directions

The future utility of this QSP framework depends on strategically addressing the challenges outlined previously. Several interconnected research directions show particular promise for advancing the field. The integration of artificial intelligence and machine learning represents a natural next step for enhancing model robustness and trustworthiness. Beyond pattern recognition, machine learning techniques can enable automated parameter estimation and, more importantly, improved uncertainty quantification, which helps to define the boundaries of model credibility (44). Furthermore, the dynamic integration of real-world data and the incorporation of single-cell technologies, such as scRNA-seq, will be crucial for validating models against real-world evidence and for defining molecular patient subtypes, thereby enabling the creation of more biologically grounded virtual populations (45, 46).

Improving model granularity and biological fidelity remains essential. Deepening the systems immunology foundation through more detailed mechanistic networks of immune cell interactions and tissue-specific microenvironments will enhance predictive accuracy for complex autoimmune pathways (47). The development of “digital twins”, highly personalized models of individual patients, constitutes a long-term vision that could revolutionize personalized therapy for heterogeneous diseases (48).

Establishing a realistic path toward standardization and accessibility, embodied by the “AutoQSP” concept, is critical for overcoming practical barriers related to reproducibility, communication, and adoption. This should evolve into a community-driven initiative involving several key components: developing shared, modular model repositories with standardized formats such as CellML and SBML accompanied by clear documentation (49); creating user-friendly software platforms with graphical interfaces that enable clinical researchers to utilize pre-built, disease-specific QSP modules without requiring extensive programming expertise (50); and establishing best-practice guidelines for model development, validation, and reporting, potentially under the guidance of regulatory agencies and professional societies (51, 52).

A particularly compelling future application involves supporting the design of N-of-1 trials for patients with ultra-rare diseases or highly unique clinical presentations (53, 54). In such cases, where traditional clinical trials are not feasible, the QSP framework can be instantiated for a single individual, creating a personalized “digital twin” through integration of the patient’s multi-omics, clinical, and biomarker data (55, 56). This model can be used in silico to test various therapeutic hypotheses by simulating different drug mechanisms, doses, and sequences, thereby identifying the optimal personalized treatment strategy with the highest predicted efficacy and safety profile prior to initiating a real-world N-of-1 trial and systematically enhancing the success rate of interventions in the most challenging clinical scenarios (57).

## 5 Conclusion

Quantitative systems pharmacology represents a promising but underutilized tool in the development of therapies for organ-specific autoimmune and rare diseases. By offering a structured, mechanistically grounded approach to trial design, QSP can enhance the precision and efficiency of clinical research. Despite challenges related to data sparsity and model validation, continued advances in computational modeling, data integration, and collaborative research can unlock the full potential of QSP. We advocate for broader adoption of QSP frameworks in early-phase clinical trials and encourage regulatory and industry stakeholders to support its development as a core component of rational drug development in complex disease settings.
